# Soybean and casein hydrolysates induce grapevine immune responses and resistance against *Plasmopara viticola*

**DOI:** 10.3389/fpls.2014.00716

**Published:** 2014-12-23

**Authors:** Nihed Lachhab, Simona M. Sanzani, Marielle Adrian, Annick Chiltz, Suzanne Balacey, Maurizio Boselli, Antonio Ippolito, Benoit Poinssot

**Affiliations:** ^1^Dipartimento di Scienze del Suolo, della Pianta e degli Alimenti, Università degli Studi Aldo MoroBari, Italy; ^2^Université de Bourgogne, UMR 1347 Agroécologie, Pôle Interactions Plantes Micro-organismes - ERL CNRS 6300Dijon, France; ^3^INRA, UMR 1347 Agroécologie, Pôle Interactions Plantes Micro-organismes - ERL CNRS 6300Dijon, France; ^4^Dipartimento di Biotecnologie, Università degli Studi di VeronaSan Floriano, Italy

**Keywords:** protein hydrolysates, *Plasmopara viticola*, *Vitis vinifera*, induced resistance, plant immunity, phytoalexins

## Abstract

*Plasmopara viticola*, the causal agent of grapevine downy mildew, is one of the most devastating grape pathogen in Europe and North America. Although phytochemicals are used to control pathogen infections, the appearance of resistant strains and the concern for possible adverse effects on environment and human health are increasing the search for alternative strategies. In the present investigation, we successfully tested two protein hydrolysates from soybean (*soy*) and casein (*cas*) to trigger grapevine resistance against *P. viticola*. On *Vitis vinifera* cv. Marselan plants, the application of *soy* and *cas* reduced the infected leaf surface by 76 and 63%, as compared to the control, respectively. Since both hydrolysates might trigger the plant immunity, we investigated their ability to elicit grapevine defense responses. On grapevine cell suspensions, a different free cytosolic calcium signature was recorded for each hydrolysate, whereas a similar transient phosphorylation of two MAP kinases of 45 and 49 kDa was observed. These signaling events were followed by transcriptome reprogramming, including the up-regulation of defense genes encoding pathogenesis-related (PR) proteins and the stilbene synthase enzyme responsible for the biosynthesis of resveratrol, the main grapevine phytoalexin. Liquid chromatography analyses confirmed the production of resveratrol and its dimer metabolites, δ- and ε-viniferins. Overall, *soy* effects were more pronounced as compared to the *cas* ones. Both hydrolysates proved to act as elicitors to enhance grapevine immunity against pathogen attack.

## Introduction

Grapevine plant (*Vitis vinifera*) has a surveillance system considered as the first defense line against invader pathogens, activated after signal molecule perception. These molecules may come from infectious agents or non-pathogenic microorganisms and therefore be designated as pathogen- or microbe-associated molecular patterns (PAMPs or MAMPs), respectively; they may correspond also to secondary products released during pathogen invasion, called damage-associated molecular patterns (DAMPs) (Boller and Felix, 2009; Dodds and Rathjen, 2010). These elicitors are perceived by pattern-recognition receptors, leading to the regulation of plant immunity known as MAMP-triggered immunity (MTI) (Nürnberger and Brunner, 2002; Boller and Felix, 2009), which is often characterized by rapid events occurring within the first minutes to few hours. For example, protein phosphorylation and de-phosphorylation generate diverse defense signaling events, as the triggering of mitogen activated protein kinases (MAPKs) and the modification of plasma membrane permeability (Romeis, 2001). In particular, the influx of extracellular Ca^2+^ causes specific variations of free cytosolic calcium concentrations ([Ca^2+^]_cyt_) into elicited cells, considered cardinal for defense response activation (Lecourieux et al., 2002; Vandelle et al., 2006). In addition, reactive oxygen species (ROS), which may have direct toxic effect on the pathogen, are produced. Depending on the host and the elicitor, these signals lead to specific transcriptional and metabolic modulations (Romeis, 2001; Garcia-Brugger et al., 2006), e.g., the synthesis of antimicrobial secondary metabolites as phytoalexins, and pathogenesis related (PR)-proteins as β-1,3 glucanases and chitinases (Garcia-Brugger et al., 2006; Van Loon et al., 2006). Key signal molecules such as salicylic acid (SA) and jasmonic acid (JA) are also produced within hours after elicitor perception and participate in the regulation of downstream defense genes (Robert-Seilaniantz et al., 2011). The stimulation prior infection of these defense responses confers a protection to the whole plant, thus might be considered as a durable and acceptable alternative to pesticide application in grapevine disease management (Ippolito and Sanzani, 2011). In fact, grapevine is highly susceptible to various diseases including downy mildew caused by *Plasmopara viticola* and gray mold caused by *Botrytis cinerea*, both diseases are responsible for yield and quality losses in most of the world's vineyards (Sanzani et al., 2012).

The efficiency and mode of action of several elicitors in triggering grapevine defense responses have been extensively studied. Bacterial elicitors, such as harpin and flagellin, proved to stimulate grapevine innate immunity through early signaling event activation and defense gene induction (Qiao et al., 2010; Chang and Nick, 2012; Trdá et al., 2014). Similarly, various fungal elicitors, such as chitin derivatives and endopolygalacturonase1 from *B. cinerea* (BcPG1), proved to enhance efficiently grapevine defense responses (Poinssot et al., 2003; Aziz et al., 2006; Vandelle et al., 2006). Finally, the β-glucan laminarin, even in its sulphated form, proved to induce various defense events in grapevine cell suspensions, including calcium influx, oxidative burst, activation of MAPKs, defense gene expression, and phytoalexin accumulation, providing protection of grapevine plantlets against downy mildew and gray mold (Aziz et al., 2003; Trouvelot et al., 2008; Gauthier et al., 2014). In particular, the main grapevine phytoalexin resveratrol has a well-known antifungal activity against grapevine pathogens by inhibiting the conidia germination and mycelial growth (Adrian et al., 1997, 2012; Aziz et al., 2003).

However, elicitors applied in the field often provide highly variable protection, probably related to their composition and the complex environment/plant/pathogen interactions (Adrian et al., 2012; Delaunois et al., 2013). Nevertheless, activation of the plant immune system prior infection remains a promising approach, which needs improvement of elicitor effectiveness, investigation of new molecules and deep knowledge of their mode of action (Caillot et al., 2012).

In the past two decades, increasing attention has been paid to the bioactive role of protein hydrolysates manufactured from various sources (Clemente, 2000). Protein hydrolysates contain a wide variety of active peptides with low molecular weight, which directly influence numerous biological processes evoking hormonal and immunological responses (Phelan et al., 2009). At present, hydrolysates manufactured from milk proteins, as casein, and leguminous plants, as soybean, are widely investigated as natural antioxidant compounds, and have interesting applications as supplements in food and pharmaceutical preparations with antimicrobial properties (Gibbs et al., 2003; Kumar et al., 2013). Besides, they proved to be as effective as a commercial repellent in preventing animals from browsing trees and shrubs (Kimball and Nolte, 2006). Finally, protein hydrolysates proved to act as biostimulants on corn (*Zea mays*), dwarf pea (*Pisum sativum*), and tomato (*Solanum lycopersicum*) (Colla et al., 2014). In previous studies, Lachhab et al. (2014, in press) reported the efficacy of soybean and casein hydrolysates in controlling gray mold of grape berries and green mold of citrus fruit. Both hydrolysates, at the tested concentrations (≥1.6 mg/ml), did not show any direct effect on the pathogen growth. These results urged us to investigate further the ability of both hydrolysates to act as elicitors of plant immunity. Therefore, a study on grapevine cell suspensions was conducted with respect to plant early signaling events, as oxidative burst, [Ca^2+^]_cyt_ variations, MAPK activation, expression of defense-related genes, and phytoalexin accumulation. The protective effect of these hydrolysates against *P. viticola* was also tested on grapevine leaves.

## Materials and methods

### Plant and cell culture materials

Grapevine plantlets (*V. vinifera* cv. Marselan) susceptible to *P. viticola*, were grown in glasshouse conditions, as described by Gauthier et al. (2014). Wild-type and apoaequorin-transformed grapevine cells (*V. vinifera* cv. Gamay) were cultivated as described by Vandelle et al. (2006).

### Elicitor preparation

Protein hydrolysates, from soybean (*soy*) and casein (*cas*), produced for the pharmaceutical and food industry were supplied by “A. Costantino & C. S.p.A.” (Favria, Turin, Italy). Since the two products are not yet on the market and protected by a trademark, their physicochemical characteristics cannot be reported. Solutions at 1.6 mg/ml in sterile ultra-pure or distilled water were prepared 24 h before use, 0.20 μm filtered and stored at 4°C. This concentration was selected based on previous trials (Lachhab et al., 2014, in press). Oligogalacturonides (OG) were provided by Goëmar laboratories (Saint-Malo, France).

### Plant treatment and inoculation

Both faces of leaves of 8-weeks old grapevine plantlets were sprayed by *soy* and *cas* hydrolysates. A volume of 10 ml per plant was used. Plant treated by water served as a control. For inoculation, *P. viticola* sporangia were collected from sporulating leaves and suspended in distilled water at a concentration of 10^4^ sporangia/ml. Inoculation was performed by spraying a freshly prepared sporangia suspension onto the abaxial face of leaves 24 h post treatment (hpt). Plants were placed for 5 h in a humid chamber (100% relative humidity, 24 ± 2°C), and then transferred back to the greenhouse. Five days post inoculation (dpi), plants were placed again in the humid chamber overnight to stimulate sporulation. Disease was assessed estimating the leaf surface area (%) covered by sporulation. Five plants were used per condition, evaluating three leaves per plant that were marked before treatment.

### Cell preparation for treatments

To investigate early signaling events (ROS, Ca^2+^ and MAPK), cells were transferred in the M10 buffer (10 mM MES, 175 mM mannitol, 0.5 mM K_2_SO_4_, 0.5 mM CaCl_2_; pH 5.3), re-suspended at 0.1 g of fresh weight of cells (FWC) per ml and equilibrated for 1 h in light (130 rpm at 24°C) before treatments (Trdá et al., 2014). For phytoalexin and gene expression analyses, they were aseptically subcultured and equilibrated at the exponential growth phase 12 h before elicitation. In each analysis, cell suspensions were treated with 1.6 mg/ml *soy* or *cas*. Water and 0.5 mg/ml OG served as negative and positive control, respectively.

### Analysis of the variations in free cytosolic calcium concentration ([Ca^2+^]_cyt_)

Variations in [Ca^2+^]_cyt_ were analyzed following aequorin bioluminescence of 250μl of apoaequorin-expressing cells using a luminometer (Lumat LB9507, Berthold Technologies, Bad Wildbad, Germany) as described in Dubreuil-Maurizi et al. (2010) with minor modifications. *In vivo* reconstitution of aequorin was performed adding 5μl of coelenterazine (5 mM stock solution in DMSO) to 5 ml of aequorin-transformed cell suspension for at least 3 h in the dark. Aequorin was quantified adding 300 μl of lysis buffer (2 M CaCl_2_; 20% ethanol, v/v). Finally, variations in [Ca^2+^]_cyt_ were calculated following the calibration equation developed by Rentel and Knight (2004).

### H_2_O_2_ production measurement

H_2_O_2_ production was determined using chemiluminescence of luminol as described in Gauthier et al. (2014). Briefly, aliquots (250 μl) of cell suspension were added with 300 μl of H50 medium (50 mM Hepes, 175 mM mannitol, 5 mM CaCl_2_, 0.5 mM K_2_SO_4_; pH 8.5) and 50 μl of 0.3 mM luminol. Relative luminescence was recorded within a 10-s period using a luminometer (Lumat LB 9507, Berthold Technologies, Bad Wildbad, Germany) and expressed as nmol H_2_O_2_ per gram of FWC.

### Western blot analyses

Western blot analyses were performed as described by Trdá et al. (2014). Aliquots containing 15 μg of protein were solubilised in a buffer (Laemmli, 1970) and submitted to 10% SDS-PAGE, before transfer to nitrocellulose membrane (Hybond ECL, Amersham Biosciences, Munchen, Germany) for western blotting. Phosphorylated MAPKs were detected with an antibody raised against a synthetic phospho-Thr202/Tyr204 peptide of human phosphorylated extracellular regulated protein kinase 1/2 (α-pERK1/2, Cell Signaling, Danvers, MA, USA). Probing and detection were performed by an ECL Western detection kit (Amersham Biosciences, Little Chalfont, UK).

### Cell death in grapevine cell suspensions

Dead cells were quantified according to Binet et al. (2001). Briefly, 24 h post treatment (hpt), cells were incubated 15 min with the vital neutral red dye 0.01%, and then washed extensively with buffer M10 (pH 7) to remove excess dye. A colorless vacuole assessed by microscope observation was considered as measure of cell death.

### Gene expression by real-time quantitative PCR (qPCR) analyses

Aliquots (2 ml) of treated cells were harvested by filtration on GF/A filters following a time course (0, 4, 8, 12, 24, 36, 48, and 72 hpt), frozen and ground in liquid nitrogen. Total RNA isolation was obtained by Trizol (Sigma-Aldrich, St. Louis, MO, USA). The RNA yield and quality were determined by NanoDrop 2000 (Thermo Scientific, Waltham, MA, USA). cDNAs were synthesized by reverse-transcribing 400 ng of total RNA using Superscript III reverse transcriptase kit (Life Technologies, Saint Aubin, France). Amplifications were run in a 96 well-plates iCycler iQ thermal cycler (BioRad, Hercules, CA, USA) and quantification was performed with the iCycler iQTM associated software (Real time Detection System Software, version 3.0). Differential expression was determined according to the 2^−ΔΔCt^ method (Livak and Schmittgen, 2001). Elongation factor EF1γ was used as a reference gene (Dufour et al., 2013). The sequences of the primer pairs used are reported in Table S1. Reaction conditions were those reported by Dubreuil-Maurizi et al. (2010). Each reaction was performed in 20 μl-volume containing 10 μl SsoAdvanced SYBR Green Supermix (BioRad), 0.5 μl (500 nM) of each primer, 1 μl of nuclease-free water and 8 μl cDNA (diluted 40-fold). For each primer pair, previously, calibration curves were built up amplifying cDNAs synthesized from serial dilutions (from 1 to 1000 ng) of total RNA. Linear equations, determination coefficients (R^2^) and reaction efficiencies were calculated.

Ct values and RNA concentrations proved to be linearly correlated with determination coefficients (R^2^) ranging from 0.94 to 0.99 (Table S2). Reaction efficiencies were similar to that of the housekeeping *EF1 γ* gene and included in the optimal range 90–110%.

### Phytoalexin quantification

After cell treatments, phytoalexins were quantified in two different fractions (cells/culture medium) separated by filtration on GF/A filters following the same time course of qPCR analyses. The culture medium samples were directly analyzed, whereas cell samples were stirred vigorously and incubated in 2 ml absolute ethanol for 24 h at 4°C to extract stilbenes. Samples were analyzed by an Acquity UPLC system (Waters, Milford, MA, USA) equipped with a model 2996 photo-diode array detector, as described by Boutegrabet et al. (2011). Each sample (10 μl) was loaded onto a BEH C18 column (Waters, Eschborn, Germany) equilibrated with water-acetonitrile-formic acid 100:10:0.1 (v/v) (Solvent A) and acetonitrile (Solvent B). Stilbenes were eluted with a stepwise gradient as follows: 0% B (0–1.73 min), 16% B (1.73–2.73 min), 42% B (2.73–4.12 min), 0% B (5–6 min) at a flow rate of 0.8 ml/min. Quantification of stilbenes was performed by calibration curves, using the peak area of different amounts of standards detected at 305 nm and 320 nm, and the chromatographic characteristics were calculated using Waters Empower software.

### Statistical analyses

When more than two conditions were present (water control, *soy* and *cas*), data were subjected to ANOVA (One-Way analysis of variance). Significant differences (*P* ≤ 0.05) were identified by the General Linear Model (GLM) procedure with the Duncan's Multiple Range Test (DMRT). Percentage data of incidence of decay underwent arcsine-square-root transformation before ANOVA analysis. For gene expression data, since just two conditions were compared (relative expression by *soy* and *cas*), Student's *t*-test was applied. Data were processed using the software package Statistics for Windows (StatSoft, Tulsa, OK, USA).

## Results

### Soybean and casein hydrolysates induce grapevine resistance against *P. viticola*

**Figure 1:**
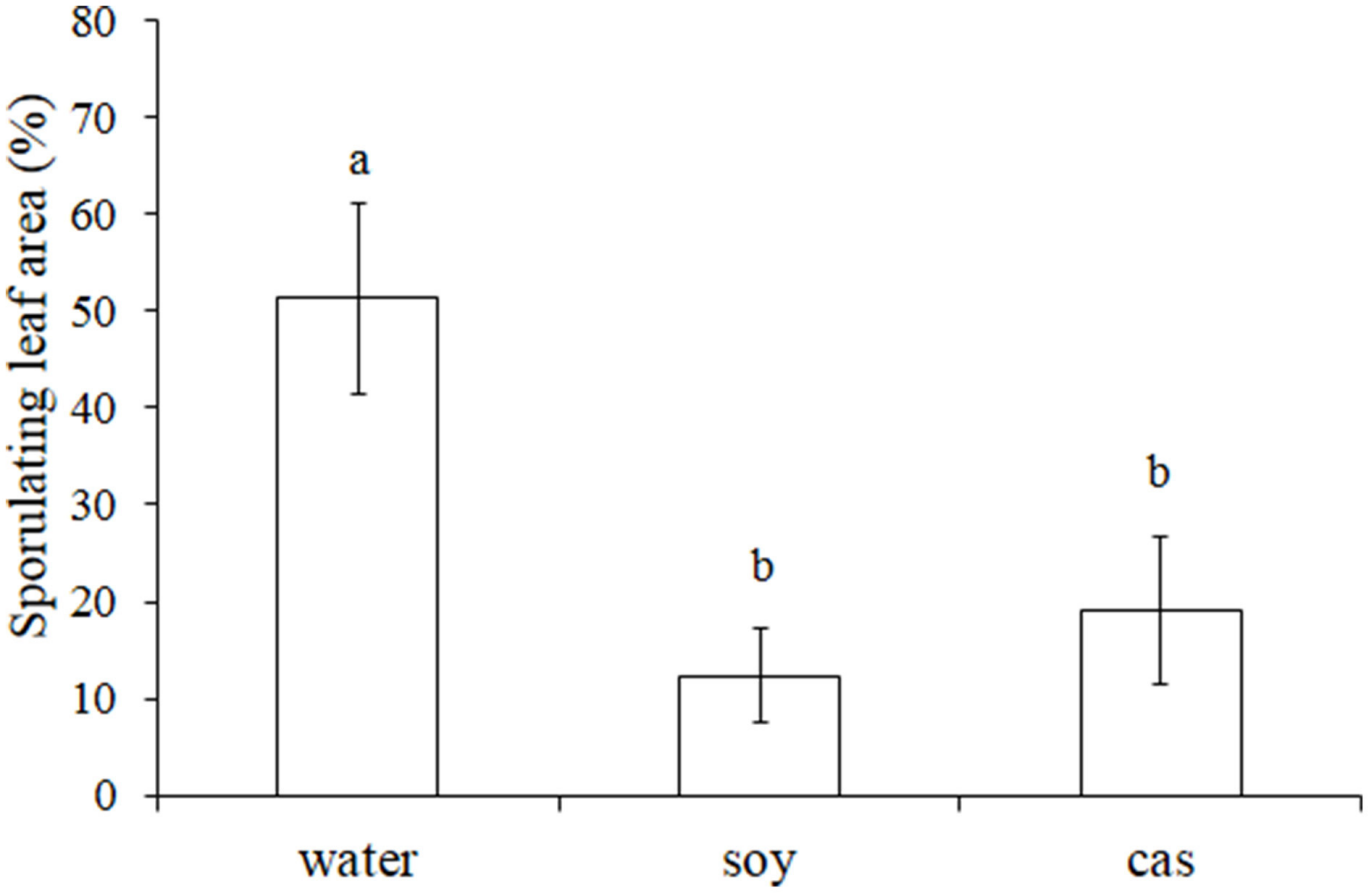
**Soybean (*soy*) and casein (*cas*) hydrolysates induce resistance of grapevine against *Plasmopara viticola***. Plantlets were sprayed with water, *soy* or *cas* (1.6 mg/ml) 24 h before *P. viticola* inoculation (10^4^ spores/ml). Leaf sporulating area (%) was evaluated at 5 DPI. Data represent means ± standard deviation from 3 leaves of 5 independent plants per modality (*n* = 15). Results are from one representative experiment out of 3 repetitions with similar results. Significant differences (*P* ≤ 0.05) were identified with the Duncan's Multiple Range Test. Bars with different letters are significantly different.

To evaluate the resistance of hydrolysates-treated grapevine plants to downy mildew, a first dose-response experiment has been realized with 0, 0.4, 0.8 and 1.6 mg/ml. Results indicated that only the 1.6 mg/ml concentration gave a reproducible plant protection against *P. viticola* (data not shown). Thereafter, plants were sprayed by *soy* and *cas* at 1.6 mg/ml, inoculated by *P. viticola* sporangia 24 h later and evaluated for the leaf sporulating area at 5 dpi. A significant reduction of disease severity by 76% for *soy* and 63% for *cas* was observed as compared to the control. However, no difference was found between the two hydrolysates (Figure 1).

### Effect of soybean and casein hydrolysates on early signaling events

To investigate whether *soy* and *cas* have the ability to act as efficient elicitors of grapevine immune responses, we characterized the early signaling events known to be triggered upon elicitor perception in cell suspensions.

**Figure 2:**
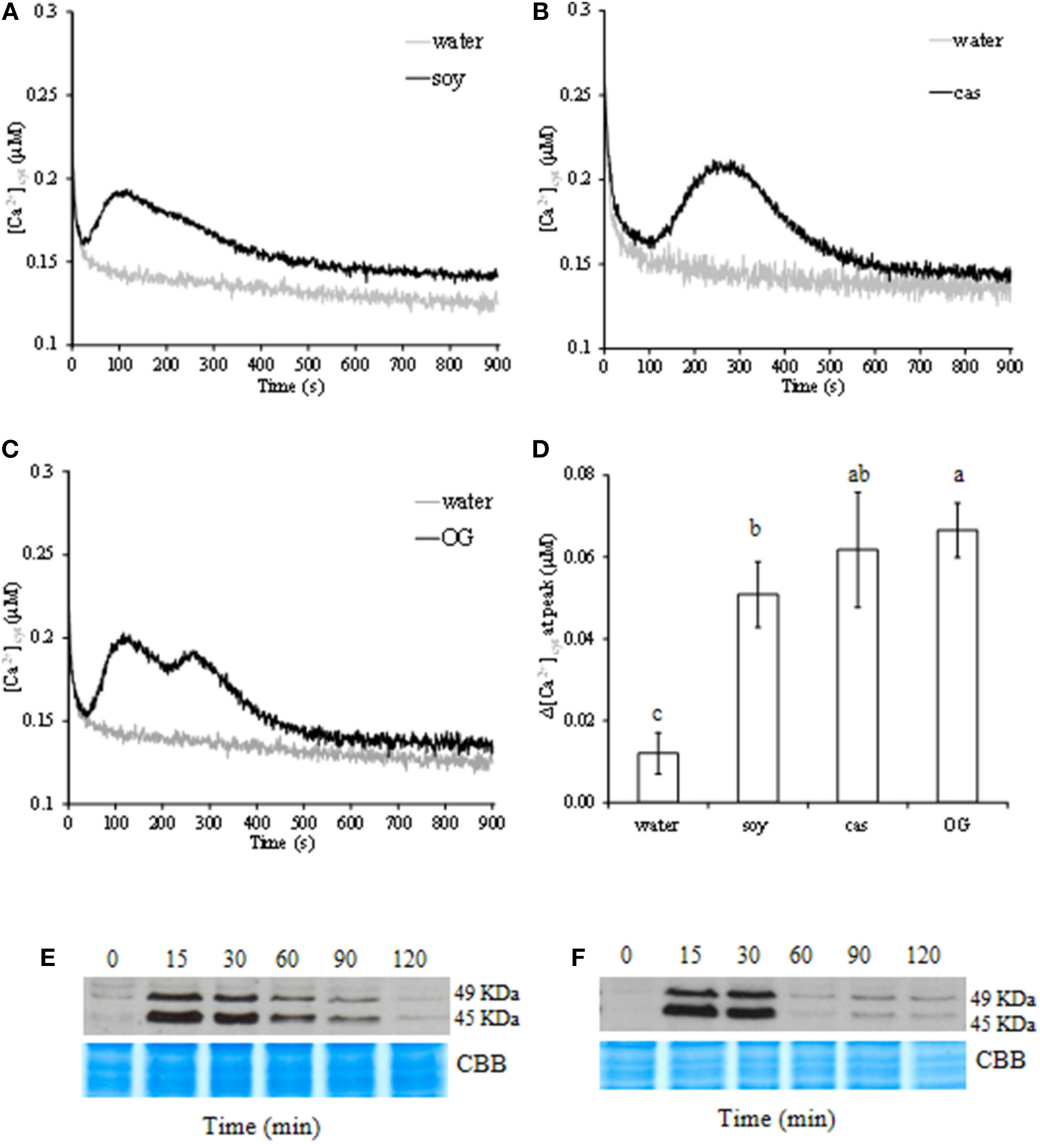
**Variations of free cytosolic calcium ([Ca^2+^]_cyt_) concentration in aequorin-expressing cell suspensions elicited by soybean (*soy*, A) and casein (*cas*, B) hydrolysate at 1.6 mg/ml, or oligogalacturonide (OG, C) at 0.5 mg/ml**. Results are from one representative experiment out of three independent ones. Quantitative representation of the difference among [Ca^2+^]_cyt_ peaks **(D)**; data represent means of three independent experiments ± standard deviation. Significant differences (*P* ≤ 0.05) were identified with the Duncan's Multiple Range Test. Bars with different letters are significantly different. Phosphorylation kinetics of two mitogen-activated protein kinases (MAPKs) of 49 and 45 kDa in **(E)** *soy*- and **(F)** *cas*-treated cells. For each treatment, homogeneous protein loading was checked by Coomassie brilliant blue staining (CBB). Results are from one representative experiment out of three.

Given the pivotal role of Ca^2+^ in transducing the signal and activating the plant surveillance system against pathogen invasion, we investigated [Ca^2+^]_cyt_ variation in elicited aequorin-expressing cells. *Soy* and *cas* derived peptides caused a transient increase in [Ca^2+^]_cyt_ with different signature in peak time and trend (Figures 2A,B). Although of similar intensity (Figure 2D), OG triggered a biphasic increase of [Ca^2+^]_cyt_ in cells (Figure 2C).

*Soy* induced a rapid increase of [Ca^2+^]_cyt_ after 40 s, peaked after 2 min, to decrease slowly without returning to the background level after 15 min as compared to OG and water (Figure 2A). Whereas in *cas*-treated cells, the level of [Ca^2+^]_cyt_ increased 1 min after treatment and picked at 4.5 min. Thereafter, [Ca^2+^]_cyt_ returned to the basal level (Figure 2B) within 10 min. When cells were treated with OG, the maximum of [Ca^2+^]_cyt_ was recorded at the first peak after 2 min, then decreased to sign the second peak at 4 min and turned back to the basal level as in *cas*-treated cells (Figure 2C).

MAPK phosphorylation was investigated by immunoblotting protein extracts from hydrolysate-treated cells with polyclonal antibodies that specifically react with the phosphorylated form of plant ERK-related activated MAPKs. *Soy* and *cas* showed ability to strongly activate two phosphorylated MAPK orthologs with molecular masses of 45 and 49 kDa (Figures 2E,F). In treated cells, the activation of protein kinases peaked after 15–30 min, and remained until 90 min in *soy*-treated cells (Figure 2E), whereas in *cas*-treated cells returned almost to the basal level within 60 min (Figure 2F).

Generation of H_2_O_2_ in cells treated with *soy* or *cas* was determined using a luminol chemiluminescence assay. A basal H_2_O_2_production was observed in control cells. The addition of OG to grapevine cells led to a rapid H_2_O_2_ production, whereas *soy* and *cas* did not cause any significant oxidative burst (data not shown).

The toxicity of *soy* and *cas* hydrolysates was also evaluated by cell viability assays, in which the number of red colored grapevine cells was assessed at 24 hpt. We did not recorded any toxic effect of the two hydrolysates compared to the water control (data not shown).

### Soybean and casein hydrolysates induce defense-related gene expression in grapevine cell suspensions

**Figure 3:**
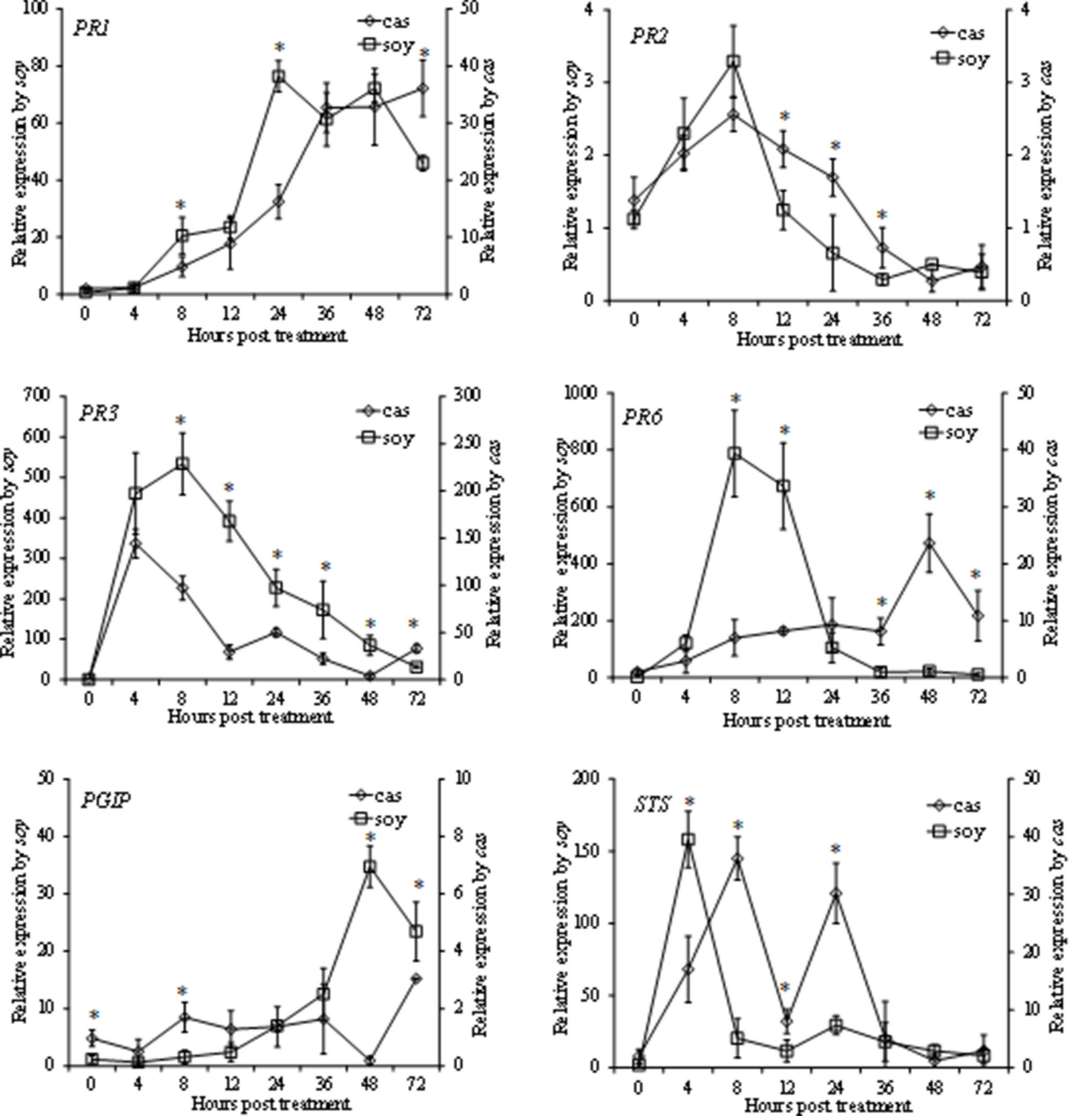
**Relative expression kinetics in grapevine cells elicited by soybean (*soy*) and casein (*cas*) hydrolysates at 1.6 mg/ml of 6 defense related genes encoding: pathogenesis-related protein 1 (*PR1*), ß-1,3-glucanase (*PR2*), chitinase 4c (*PR3*), protease inhibitor (*PR6*), polygalacturonase-inhibiting protein (*PGIP*) and stilbene synthase (*STS*)**. Cell samples were collected at 0, 4, 8, 12, 24, 36, 48, 72 h post treatment. Water-treated cells were used as a control. Data were analyzed with the 2^-ΔΔCt^ method using data of water treated cells as a control, and normalized using the *EF1γ* housekeeping gene. Data represent the means of three experiments ± standard deviation. Statistical significances (*P* ≤ 0.05) were determined using Student's *t*-test using paired data. ^*^ indicates significant differences between treatments. Since, in presence of the two treatments, highly different expression extents were recorded for most of the genes, two Y-axes were used in the graphs. Relative expression values in presence of each treatment refer to the respective axis.

In this study we evaluated by qPCR in time-course experiments the expression in grapevine cell suspensions of six selected defense genes known to be activated in response to various elicitors: those encoding a pathogenesis-related protein 1 (*PR1*), a ß-1,3-glucanase (*PR2*), a chitinase 4c (*PR3*), a protease inhibitor (*PR6*), a polygalacturonase-inhibiting protein (*PGIP*) and a stilbene synthase (*STS*). Overall, *soy* and *cas* induced a rapid and high transcript accumulation of most of the tested genes with different kinetics and intensities; a more pronounced effect of *soy* was generally observed (Figure 3). With the exception of *PR1*, whose transcript accumulation was up-regulated late in both hydrolysate-treated cells, the expression pattern of genes encoding the antifungal proteins *PR2* and *PR3* were similar in grapevine cells treated with both hydrolysates, with rapid and massive accumulation of *PR3* transcripts in *soy*-elicited cells, which reached a 534-fold increase at 8 hpt. The latter hydrolysate induced a similar accumulation also of *PR6*, that peaked at 8 hpt by 790-fold, and remained more expressed than in water-treated cells until 72 hpt. When treated with *cas*, the *PR6* expression pattern showed a different trend, reaching the maximum by 24-fold only at 48 hpt. Concerning *PGIP*, transcript accumulations was up-regulated even later than *PR1* in both hydrolysate-treated cells, being more pronounced in *soy*-treated cells. Finally, following treatment of the grapevine cell suspensions with the two hydrolysates, the *STS* gene, encoding a key enzyme of the resveratrol biosynthesis (the main grapevine phytoalexin), showed a biphasic expression profile. It peaked at 4 or 8 hpt for *soy*- and *cas*-treated cells, respectively.

### Soybean and casein hydrolysates induce phytoalexin accumulation in grapevine cell suspensions

**Figure 4:**
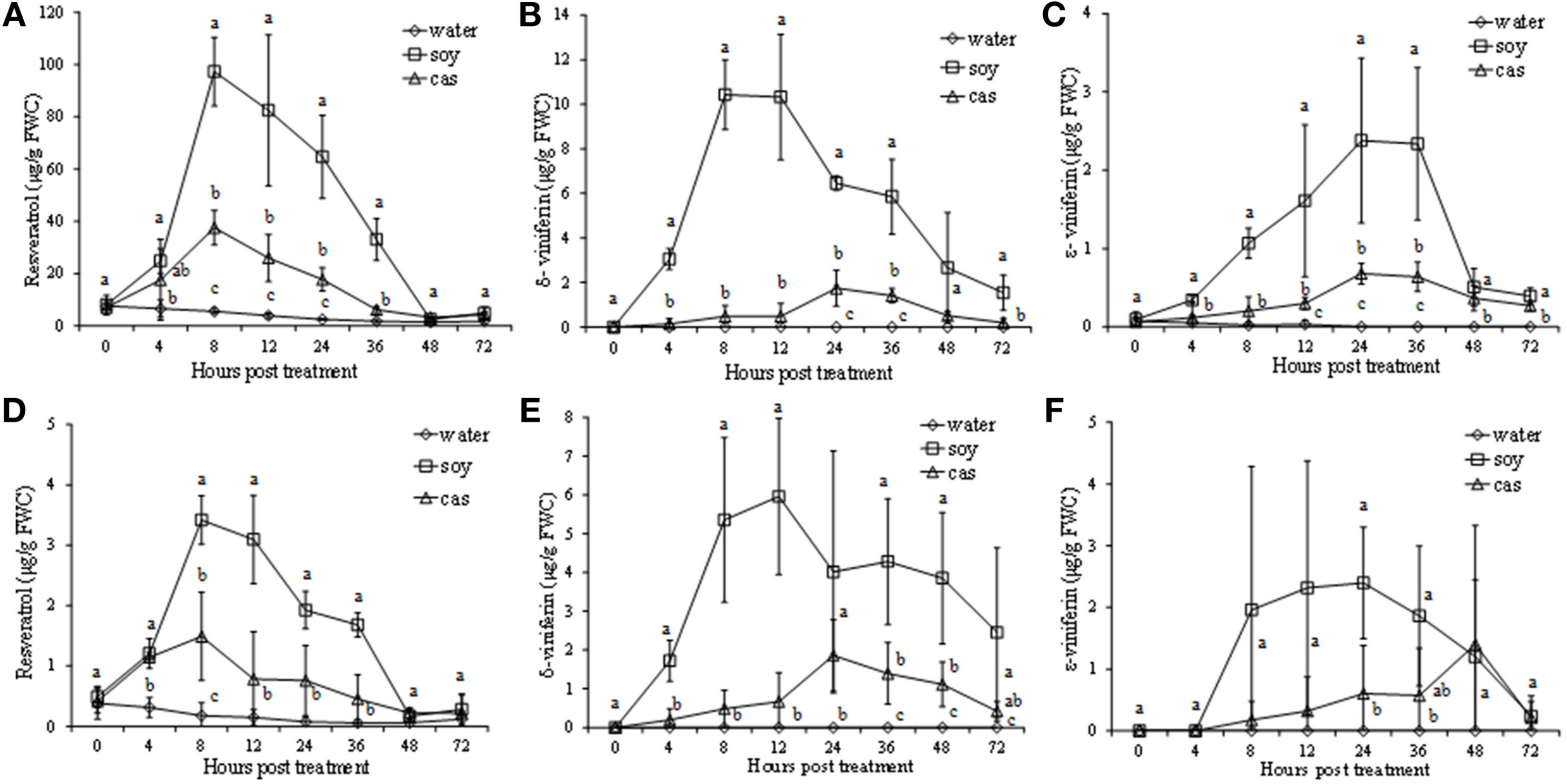
**Stilbene production in culture medium (A–C) and grapevine cells (D–F) elicited by soybean (*soy*) and casein (*cas*) hydrolysates from 0 to 72 h post treatment**. Resveratrol **(A,D)**, δ-viniferin **(B,E)**, ε-viniferin **(C,F)** amounts were expressed as μg/g of fresh weight of cells (FWC). Data represent the mean of three independent experiments ± standard deviation. Significant differences (*P* ≤ 0.05) were identified with the Duncan's Multiple Range Test. Time-points with different letters are significantly different.

Both protein hydrolysates, in particular those extracted from soybean, showed ability to elicit the production of stilbenes. Concentrations in the culture medium and inside the cells varied at different extents (Figure 4). In particular, a higher accumulation of resveratrol (3,5,4′-trihydroxystilbene) was recorded in the culture medium. It was already detected at 4 hpt, significantly increased to reach 97 and 37 μg/g FWC after 8 hpt for *soy*- and *cas*-treated cells, respectively, and then decreased to the level of water control at 48–72 hpt (Figure 4A). A similar trend, although with lower values, was observed for resveratrol inside the cells (Figure 4D).

The resveratrol dimers δ- and ε-viniferin, significantly increased after treatment by the two hydrolysates both in culture medium and inside the cells.

In culture medium of *soy*-treated cell suspension, δ-viniferin peaked at 8–12 hpt, reaching 10 μg/g FWC (Figure 4B), whereas ε-viniferin reached the maximum level at 24 hpt (2.37 μg/g FWC; Figure 4C). A less pronounced accumulation of δ-viniferin and ε-viniferin in the culture medium was recorded in the *cas*-treated cell suspension, peaking at 24 hpt (Figures 4B,C).

In cells elicited by *soy*, δ- and ε- viniferin peaked at 12 or 24 hpt with values of 6 and 2.3 μg/g FWC, respectively. In cells elicited by *cas* the two resveratrol dimers showed a low increase at 24 and 48 hpt, with values of 1.9 and 1.4 μg/g FWC, respectively (Figures 4E,F).

The resveratrol glucoside piceid was not significantly accumulated in hydrolysate-treated cells and culture medium as compared to the water control (data not shown).

## Discussion

On the bases of the effectiveness of *soy* and *cas* hydrolysates to trigger resistance against gray and green mold (Lachhab et al., 2014, in press) and downy mildew (this study) without any effect on the growth *in vitro* of the different pathogens (Lachhab et al., 2014, in press), we hypothesized that the two hydrolysates might act as general elicitors. They could trigger signaling events involved in grapevine immunity as already reported elsewhere for other elicitor molecules (Aziz et al., 2003, 2007; Poinssot et al., 2003; Trouvelot et al., 2008; Gauthier et al., 2014; Trdá et al., 2014). Therefore, we investigated their effect on grapevine cells with respect to [Ca^2+^]_cyt_ variations, H_2_O_2_ production, MAPK activation, defense genes expression, and phytoalexin production. Both *soy* and *cas* induced specific changes of [Ca^2+^]_cyt_, considered essential for defense response activation (Lecourieux et al., 2002; Tuteja, 2009). In particular, cells elicited by the two hydrolysates showed a rapid increase of [Ca^2+^]_cyt_, as compared to water control, similarly to other elicitors as OG, cryptogein, BcPG1, and Flg 22 (Lecourieux et al., 2006; Vandelle et al., 2006; Aziz et al., 2007; Trdá et al., 2014). However, *soy* and *cas* showed two specific signatures, characterized by different kinetics and duration, suggesting different active molecules in each hydrolysate (Blume et al., 2000; Knight, 2000; Lecourieux et al., 2006). In fact, in *soy*-treated cells, the increase in [Ca^2+^]_cyt_ was more rapid than that triggered by *cas*, and the level of free cytosolic calcium remained higher as compared to water and *cas* treatments. A similar behavior was observed in [Ca^2+^]_cyt_ signatures induced by the polypeptide cryptogein in tobacco cells (Lecourieux et al., 2006), although it was not observed in response to a range of different oligosaccharide elicitors, including our positive control OG. These findings suggest that the active elicitor molecule in *soy* is likely not an oligosaccharide as assumed elsewhere (Lachhab et al., in press). The characterization of *soy* and *cas* chemical composition by the manufacturer is in progress.

Calcium signaling is known to act upstream of the MAPK pathway in some plant defense responses (Link et al., 2002; Lecourieux et al., 2006). In this study, we showed in *soy*-treated cells a fast, strong, and lasting MAPK activation, which is consistent with the high level of free cytosolic calcium concentration. These results are similar to those of Lecourieux et al. (2002) who recorded an activation of MAPKs in tobacco cells at 2 h following cryptogein elicitor application. The relation between [Ca^2+^]_cyt_ and MAPK stimulation is more evident considering the *cas*- effect. Indeed, the rapid decrease of the [Ca^2+^]_cyt_ level is correlated with a very low activation of MAPKs after 60 min, as previously demonstrated in cryptogein-treated tobacco cells (Lecourieux et al., 2002).

In addition to [Ca^2+^]_cyt_ variations and MAPKs activation, many authors described the rapid release of H_2_O_2_ in cell suspensions as a response to various elicitors (Poinssot et al., 2003; Aziz et al., 2007; Trouvelot et al., 2008). However, *soy*- and *cas*-elicited cells did not show any variation in H_2_O_2_ production. Furthermore, similarly to the β–glucan derivative PS3, the two hydrolysates did not have any toxic effect on grapevine cells, supporting the hypothesis that the resistance to pathogen invasion may depend on other defense responses rapidly activated during pathogen infection (Aziz et al., 2007; Gauthier et al., 2014). In fact, in some instances, H_2_O_2_ generation is not necessary for the induction of defense reactions. As shown by Galletti et al. (2008), a null mutation of AtrbohD-mediated oxidative burst completely abolished the induction of oxidative burst elicited by OGs in *Arabidopsis*, without affecting the expression of genes involved in defense responses effective against *B. cinerea*. In addition, Pauw et al. (2004) showed that ROS generation is neither necessary nor sufficient for the induction of genes involved in the terpenoid indole alkaloids biosynthesis by yeast crude extract in *Catharanthus roseus*.

Consistently with previous reports, the two tested hydrolysates caused an up-regulation of 6 known defense-related genes (Bézier et al., 2002; Aziz et al., 2003, 2007; Trouvelot et al., 2008; Trdá et al., 2014). It has been shown that a better resistance to *P. viticola* of some *Vitis* species as *V. riparia*, was associated to a more rapid and stronger induction of defense gene expression, as compared to susceptible cultivars, e.g., *V. vinifera* (Polesani et al., 2010). Among them particularly relevant are those genes encoding enzymes involved in the phenylpropanoid pathway as STS, and PR-proteins (chitinase and β-1,3 glucanase), whose hydrolytic activities might exert an antimicrobial effect against different pathogens, and that can play themselves a role as elicitors through the release of chitin and glucan oligomers (Fritig et al., 1998; Aziz et al., 2007). In particular, the two hydrolysates elicited marker genes of SA and JA pathways, namely *PR1* and *PR6* (Lu et al., 2009; Gfeller et al., 2011). In Arabidopsis, SA signaling is classically described to trigger resistance against biotrophic pathogens, whereas JA/ET signaling activates resistance against necrotrophic pathogens (Glazebrook, 2005). Thus, the strong and rapid induction of *PR6* is in agreement with the protective effect provided by the two hydrolysates to grapevine against *Botrytis* attack (Lachhab et al., 2014). Moreover, the two hydrolysates induced the expression of *STS* the key gene of the biosynthesis of resveratrol, known to increase resistance against pathogens (Coutos-Thévenot et al., 2001; Adrian et al., 2012), e.g., controlling conidia germination and mycelial growth in *Phomopsis viticola* and *B. cinerea* and affecting *P. viticola* zoospore mobility (Adrian et al., 1997; Pezet et al., 2003).

As expected, the two protein hydrolysates, in particular *soy*, induced a high and rapid accumulation of resveratrol at 4 hpt, the first time-assessment in our trials, meaning that *STS* gene was induced even before. This finding is in agreement with the concept that stilbene is the primary inducible response of grapevine against a number of biotic and abiotic stresses (Adrian et al., 1997; Coutos-Thévenot et al., 2001; Poinssot et al., 2003).

Interestingly, resveratrol was present at high amounts in culture medium, but showed low accumulation in cells. As suggested by Adrian et al. (2012), part of the cell-excreted resveratrol is cross-linked to the cell wall, contributing to cell strengthening.

The δ- and ε-viniferin dimers, considered as important markers for resistance of grapevine to pathogens (Malacarne et al., 2011), were detected later, as compared to resveratrol, both in culture medium and inside the cells, which is in accordance with the role of resveratrol as a precursor of viniferins. In particular, δ-viniferin more strongly accumulated, as compared to ε-viniferin, in soybean treated cells at 8–12 hpt. The δ-viniferin was reported to play an important role in resistance against downy mildew and inhibit the zoospore mobility and mycelium development of *P. viticola* (Pezet et al., 2004). Indeed, resistant cultivars synthesize high levels of resveratrol after infection, which is rapidly oxidized to yield highly toxic viniferins (Pezet et al., 2004). The success rate of elicitor molecules in inducing plant resistance depends on the coordination between the different triggered defensive events, but also on the rapidity of their activation, that hang on the complex plant/elicitor/pathogen interaction (Giannakis et al., 1998). In this study, *soy* showed pronounced efficiency in inducing defense responses, as compared to *cas* treatment, in particular concerning stilbene accumulation. The different results may be ascribed to their different proteic origins. Indeed, soybean contains different proteins as lipid transfer proteins (Kido et al., 2010) and proved to be effective against gray mold also on strawberries (Romanazzi et al., 2013). Casein has an antioxidant activity and the ability to enhance calcium uptake, by forming soluble phosphopeptides activating cell receptors (Gobetti et al., 2004) and stimulating the defenses against bacteria, yeast and filamentous fungi (Fadaei, 2012). However, to better understand the mode of action of these hydrolysates, an investigation of the large number of peptides present in each hydrolysate is needed. Moreover, further specific trials are ongoing to evaluate their stability and effectiveness in the field against *P. viticola*.

In conclusion, the presented data support the use of *cas* and particularly *soy* hydrolysates as enhancers of grapevine innate immunity and thus good candidates to replace or reduce fungicide applications in modern sustainable viticulture, as they are cheap, easily available and safe to humans and the environment.

## Author contributions

Nihed Lachhab: conducted the experiments, analyzed data and prepared the manuscript; Simona M. Sanzani: designed the experiments, analyzed the data and prepared the manuscript; Marielle Adrian: supervised phytoalexin quantification, analyzed the data and edited the manuscript; Annick Chiltz and Suzanne Balacey: helped in conducting the experiments; Maurizio Boselli: helped to design the experiments and edited the manuscript; Antonio Ippolito: helped to design the experiments and edited the manuscript; Benoit Poinssot: designed the experiments, analyzed the data and prepared the manuscript. All authors have read the manuscript and agree with its content.

## Conflict of interest statement

The authors declare that the research was conducted in the absence of any commercial or financial relationships that could be construed as a potential conflict of interest.
