# Supplementary material for: Soybean and casein hydrolysates induce grapevine immune responses and resistance against Plasmopara viticola
Source: Front Plant Sci. 2014 Dec 23;5:716. doi: 10.3389/fpls.2014.00716 (PMC4274885; doi:10.3389/fpls.2014.00716)
Supplement: Supplementary file 1 [file Table1.DOCX]

**SUPPLEMENTARY MATERIAL**

**Table S1.** Primers used for gene expression analysis.

| Primers | Sequences (5’-3’) | Target genes | References |
| --- | --- | --- | --- |
| Vv_PR6 F | AGTTCAGGGAGAGGTTGCTG | Protease inhibitor | Trouvelot et al., 2008 |
| Vv_PR6 R | CGTCGACCCAAACACGGACCCTAGTGC |  |  |
| Vv_STS F | AGGAAGCAGCATTGAAGGCTC | Stilbene synthase | Trouvelot et al., 2008 |
| Vv_STS R | TGCACCAGGCATTTCTACACC |  |  |
| Vv_PGIP F | CCATCTTCTCCGGCCAGCTAT | Polygalacturonase inhibiting protein | Aziz et al., 2003 Poinssot et al., 2003 |
| Vv_PGIP R | CGAGGTTGGGGAGGAGAGAGA |  |  |
| Vv_PR3 F | GCAACCGATGTTGACATATCA | Chitinase 4c | Aziz et al., 2003  Trdá et al., 2014 |
| Vv_PR3 R | CGTCGCCCTAGCAAGTGAG |  |  |
| Vv_PR1 F | GCGTGGGTGGGGAATGCCGA | Pathogenesis related 1 | Trdá et al., 2014 |
| Vv_PR1 R | GATGTTGTCCCTGATAGTTGCC |  |  |
| Vv_PR2 F | TCAGCCGTCCTCGGCAAATCA | Glucanase | This study |
| Vv_PR2 R | TTGGCCAGGAGTGGGGAGCC |  |  |
| EF1 γ F | GAAGGTTGACCTCTGGGATG | Elongation factor γ | Dufour et al., 2013 |
| EF1 γ R | AGAGCCTCTCCCTCAAAAGG |  |  |
