# Supplementary material for: Soybean and casein hydrolysates induce grapevine immune responses and resistance against Plasmopara viticola
Source: Front Plant Sci. 2014 Dec 23;5:716. doi: 10.3389/fpls.2014.00716 (PMC4274885; doi:10.3389/fpls.2014.00716)
Supplement: Supplementary file 2 [file Table2.DOCX]

**SUPPLEMENTARY MATERIAL**

**Table S2.** Linear equations, determination coefficients (R^2^) and reaction efficiencies obtained by plotting cDNA concentrations and Ct values in qPCR reactions for each tested primer pair.

| Gene | Linear equation | R^2^ | Efficiency (%) |
| --- | --- | --- | --- |
| *PR1* | Y= -3.258X + 27.05 | 0.99 | 103 |
| *PR2* | Y= -3.370X + 34.361 | 0.95 | 98 |
| *PR3* | Y= -3.107X + 26.260 | 0.99 | 109 |
| *PR6* | Y= -3.299X + 27.058 | 0.99 | 101 |
| *PGIP* | Y= -3.237X + 23.348 | 0.99 | 103 |
| *STS* | Y= -3.298X + 25.085 | 0.96 | 101 |
| *EF1 γ* | Y= -3.245X + 28.97 | 0.94 | 103 |
